# Looks like SNARC spirit: Coexistence of short- and long-term associations between letters and space

**DOI:** 10.1177/17470218251324437

**Published:** 2025-02-17

**Authors:** Lilly Roth, Julia F. Huber, Sophia Kronenthaler, Jean-Philippe van Dijck, Krzysztof Cipora, Martin V. Butz, Hans-Christoph Nuerk

**Affiliations:** 1 Department of Psychology, University of Tübingen, Tübingen, Germany; 2 Department of Applied Psychology, Thomas More University of Applies Sciences, Antwerp, Belgium; 3 Department of Experimental Psychology, Ghent University, Ghent, Belgium; 4 Centre for Mathematical Cognition, Loughborough University, Loughborough, UK; 5 Department of Computer Science, University of Tübingen, Tübingen, Germany; 6 LEAD Graduate School & Research Network, University of Tübingen, Tübingen, Germany; 7 German Center for Mental Health (DZPG), Berlin, Germany

**Keywords:** SNARC effect, letters, working memory, Ordinal Position Effect, Spatial-Alphabetical Association of Response Codes, SAARC effect

## Abstract

Many studies have demonstrated spatial-numerical associations, but the debate about their origin is still ongoing. Some approaches consider cardinality representations in long-term memory, such as a Mental Number Line, while others suggest ordinality representations, for both numerical and non-numerical stimuli, originating in working or long-term memory. To investigate how long-term memory and working memory influence spatial associations and to disentangle the role of cardinality and ordinality, we ran three preregistered online experiments (*Ntotal* = 515). We assessed spatial response preferences for letters (which only convey ordinal but no cardinal information, in contrast to numbers) in a bimanual go/no-go consonant-vowel classification task. Experiment 1 (“no-go” trials: non-letter symbols) validated our setup. In Experiments 2 and 3, participants learned an ordinal letter sequence prior to the task, which they recalled afterwards. In Experiment 2, this sequence was merely maintained (“no-go” trials: non-letter symbols), whereas in Experiment 3, it needed to be retrieved during the task (“no-go” trials: letters outside the sequence). We replicated letter-space associations based on the alphabet stored in long-term memory (i.e., letters earlier/later in the alphabet associated with left/right, respectively) in all experiments. However, letter-space associations based on the working memory sequence (i.e., letters earlier/later in the sequence associated with left/right, respectively) were only detected in Experiment 3, where retrieval occurred during the task. Spatial short- and long-term associations of letters therefore seem to coexist. These findings support a hybrid model that incorporates both short- and long-term representations, which applies similarly to letters as to numbers.

Numbers play a fundamental role in many contexts in humans’ everyday life. How humans mentally represent and process them is therefore a fundamental research question within the field of cognition. In 1993, Dehaene and colleagues observed what became widely known as the Spatial-Numerical Association of Response Codes (SNARC) effect. This association of small numbers with the left side and of large numbers with the right side has been demonstrated in many experiments, at least for Western cultures. The SNARC effect could be found in different tasks (see Wood et al., 2008 for a meta-analysis) and with different stimuli (e.g., digits, number words in visual or auditory modality or dice patterns; Nuerk et al., 2005). Most commonly, the effect is investigated with classification tasks like parity judgement (where participants classify numbers as even or odd; e.g., Dehaene et al., 1993) or magnitude classification (where participants classify a number as smaller or larger than a given reference number; e.g., Dehaene et al., 1990). Interestingly, the SNARC effect also occurs in tasks where the number magnitude is task-irrelevant, meaning it is not necessary to access the number magnitude information to solve the task correctly (such as in parity judgement, Dehaene et al., 1993, or font colour discrimination, Roth et al., 2025a). This has been interpreted as evidence for an automatic processing of number magnitude (Fias et al., 2001; Gevers et al., 2003).

## Spatial associations of quantity and ordinality

Not only number magnitude but also non-numerical quantities (e.g., physical size or luminance; Cohen Kadosh et al., 2008; Fumarola et al., 2014; Ren et al., 2011) can be associated with space. These observations are accounted for by a Theory of Magnitude (ATOM; Bueti & Walsh, 2009; Walsh, 2003). Moreover, well-known ordinal sequences might also be related to space (e.g., letters of the alphabet; Gevers et al., 2003). According to Casasanto and Pitt (2019), referring to the ordinal position of objects in a sequence rather than to their quantity is sufficient to explain spatial mappings such as the SNARC effect. In contrast, Prpic et al. (2016) proposed that mechanisms underlying the SNARC effect depend on whether a *direct* task (i.e., magnitude- or order-relevant instructions, like magnitude classification) or an *indirect* task (i.e., magnitude- or order-irrelevant instructions, like parity judgement) is used (see also Prpic et al., 2021). This conclusion was derived from direct (bimanual note-value comparison) and indirect (unimanual note detection) tasks with musical note values, where order (usually starting from the whole note followed by the half note, the quarter note, the eight note, etc.) is opposed to magnitude (starting from the shortest duration and ending with the whole note). Thus, while mainly ordinal information drives the SNARC effect in direct tasks, magnitude information drives the SNARC effect in indirect tasks more than ordinal information does (see Cipora et al., 2020; Schroeder et al., 2017a, for integrative views).

Interestingly, spatial mappings have been observed both for arbitrary sequences trained within an experiment and stored in working memory (WM; e.g., van Dijck & Fias, 2011) as well as for well-known ordinal sequences stored in long-term memory (LTM; e.g., months and letters in Gevers et al., 2003; well-trained but arbitrary lists of objects in Previtali et al., 2010). The order of an overlearned sequence and the order of a sequence trained within an experiment can be pitted against each other to see which of them is decisive for spatial mapping (Koch et al., 2023). Importantly, using non-numerical stimuli with an overlearned order, one can distinguish between the spatial mapping of LTM and WM ordinality, while entirely excluding magnitude information. Given that previous results for direct tasks speak for the role of ordinality, we focus here on an indirect task. In this article, when we speak of *direct* or *indirect* tasks with non-numerical stimuli, we refer to whether participants are explicitly asked to process ordinality or not, independent of whether the long-term ordinality (i.e., canonical order stored in LTM, e.g., the alphabetical order for letters) or short-term ordinality (i.e., ordered sequence stored in WM) is meant (see differentiation in the literature in the online Supplementary Material C, Table C1).

Although Prpic et al. (2016) postulate that cardinality is more decisive than ordinality for the spatial mapping of numbers in indirect tasks, the spatial mapping of ordinality stored in LTM has been observed in indirect tasks with other stimuli (e.g., consonant-vowel judgement; Gevers et al., 2003, Exp. 2). A spatial mapping of ordinality has been also observed for WM content; however, to our understanding, despite being indirect, previous studies showing a WM-dependent mapping were making the WM demand salient to the participants (e.g., by requiring a constant retrieval of the WM content while performing the indirect task). Importantly, the spatial mapping of ordinality coming from WM may depend on the salience of this sequence stored in WM (i.e., how prominent the WM content is during the task to be performed), although the ordinality within this sequence might not be task-relevant or salient. Finding a spatial association in an indirect judgement task, where neither processing cardinality nor ordinality is required, can be seen as stronger evidence for the significant role of ordinality in spatial mental representations. To disentangle spatial associations consistent with ordinal information stored in WM vs. LTM, we investigated spatial associations of letters (which convey ordinal but not cardinal information, unlike numbers) in indirect tasks and manipulated how salient the retrieval of sequences stored in WM were during the task.

## The role of WM and LTM ordinality for the SNARC and SNARC-like effects

The question arises as to how spatial associations of numbers or other kinds of stimuli with cardinality or ordinality properties can be explained. In their seminal article, Dehaene et al. (1993) described different explanations for the origins of spatial-numerical associations that are still part of the debate today: The SNARC effect is either based only on the representation of number magnitude / quantity (i.e., cardinality), or it is the result of an overlearned sequential structure of the stimuli (i.e., ordinality). Since Dehaene et al. (1993; Experiment 4) found no response preference for letters, they concluded that the SNARC effect arises from the magnitude representation, which can be imagined as a left-to-right oriented Mental Number Line (MNL; Moyer & Landauer, 1967; Restle, 1970). Note that Dehaene et al. (1993) only tested 10 participants in their consonant-vowel classification task (Experiment 4), so a true underlying effect might just have remained undetected due to poor statistical power for small effects. This is plausible, as the SNARC effect for Arabic digits in parity judgement has been shown not to be present in each individual and not to be stable over time within individuals (Roth et al., 2024). Crucially, Gevers et al. (2003, 2004) showed spatial response preferences for various non-numerical stimuli, namely letters, months, and days of the week, in both direct (e.g., order-relevant judgements on whether a letter comes before or after the letter O in the alphabetical order) and indirect (e.g., order-irrelevant judgements on whether the letter is a consonant or a vowel) task instructions. This effect has been called the Spatial-Alphabetical Association of Response Codes (SAARC) effect because letters early/late in the alphabet were associated with the left/right, respectively). Accordingly, the explanation of the SNARC effect being the result of the sequential structure of the stimuli (i.e., the inherent ordinal aspect of numbers) rather than an underlying spatially oriented MNL cannot be ruled out.

Van Dijck and Fias (2011) developed a theory that can explain spatial associations of numerical and non-numerical stimuli with ordinality, like letters or months (Gevers et al., 2003) or newly memorised arbitrary sequences of words or symbols (Previtali et al., 2010; van Opstal et al., 2009). Instead of assuming a stable spatial long-term representation of numbers in the form of a MNL, they postulated the involvement of WM in the emergence of spatial-numerical associations (i.e., the WM account; van Dijck et al., 2014; see Abrahamse et al., 2016, for later theoretical developments). Specifically, they assume that the SNARC effect is a result of the ordinal position of the elements stored in WM such that stimuli at the beginning of a sequence are associated with the left and stimuli at the end of a sequence are associated with the right (e.g., ordered sequences of numbers, but also of fruits and vegetables, as in van Dijck and Fias, 2011).

This WM account is supported by two empirical findings: First, WM seems to be involved in the emergence of the SNARC effect, as the SNARC effect disappeared under WM load depending on the task and the type of WM load. Namely, the SNARC effect disappears under verbal WM load in parity judgement (i.e., indirect task) and under spatial WM load in magnitude classification (i.e., direct task; Herrera et al., 2008; van Dijck et al., 2009). These findings demonstrate that WM capacity is required for the SNARC effect to arise, although the manifestation of this effect depends on the type of information held in WM (i.e., verbal or spatial). Second, the WM account is supported by the observation that the association of numbers and space is very flexible and varies depending on task demands (for an overview about situated influences on spatial-numerical associations, see Cipora et al., 2018). Some exemplary findings speaking in favour of the WM account are that the spatial associations depend only on the relative number magnitude within the used number range (Dehaene et al., 1993; Fias et al., 1996; but see Roth et al., 2025b), the impact of the number context (e.g., ruler vs. clock) imagined prior to the task (Bächtold et al., 1998; but see also Mingolo et al., 2024), or the location of numbers in a text (Fischer et al., 2010). These findings cannot be explained with a stable long-term association between space and numbers but rather suggest the involvement of WM. The spatial-numerical associations are assumed to be due to the position of the numbers (or any other stimuli) in WM and, thus, are rather spatial-positional associations (van Dijck & Fias, 2011).

To test their account, van Dijck and Fias (2011) used a thoughtful design, which combined the typical parity judgement task with a go/no-go paradigm, where the go/no–go classification relates to a previously learned sequence. Participants had to memorise a randomly generated sequence of five numbers between 1 and 10. Afterwards, they performed parity judgement, but only for numbers that were part of the memorised number sequence (“go” stimuli). Numbers that were not part of the memorised number sequence were not to be judged (“no-go” stimuli). After the task, it was checked whether participants had correctly memorised the sequence of numbers and only those blocks were included in subsequent analyses. Van Dijck and Fias (2011) observed that the spatial response preferences no longer relate to the number magnitude (i.e., no SNARC effect) but to the position of the numbers within the memorised number sequence. This phenomenon has been referred to as the Ordinal Position Effect (OPE; Ginsburg et al., 2014; which is the term we will use in the current manuscript) or as SPoARC (Spatial-Positional Association of Response Codes) effect (Guida & Lavielle-Guida, 2014).

In their recent opinion article, Abrahamse and van Dijck (2023) argue that ordinality eliciting the OPE refers to the serial position of an item in a so-called ranking-space relative to other items in that ranking-space. If no specific manipulation is implemented, the set of the stimuli used in the study is encoded as such a sequence (i.e., numbers from 1 to 9 in the case of most SNARC studies). Importantly, when testing whether spatial response preferences are driven by magnitude or by ordinality, it is crucial to differentiate between ordinality based on short-term representations (i.e., newly learned arbitrary sequences stored in WM) or long-term representations (i.e., overlearned ordered sequences stored in LTM).

In Experiment 2, van Dijck and Fias (2011) were able to replicate the OPE using non-numerical stimuli. Importantly, the manipulation also worked with learned sequences of fruits and vegetables, which do not have any inherent order (as compared to letters, months, and days of the week). Ginsburg et al. (2014) replicated the results of van Dijck and Fias (2011, Exp. 1) using number stimuli, ruling out that the presence of the OPE and the absence of the SNARC effect was due to specific aspects of the design like the task (parity judgement or magnitude classification) or the response-to-key assignment (switch within or between participants).

Importantly, Ginsburg et al. (2014) found that the retrieval of the memorised sequence during the classification task (as in their Experiments 1 and 2 but not 3 and 4) is necessary to observe an OPE. In van Dijck and Fias’ (2011) design the retrieval of the memorised sequence during the indirect classification task was ensured by the go/no-go design, as in the classification phase participants had to respond only to numbers that were part of the memorised sequence (i.e., high WM salience). When participants were instructed to respond to all numbers independently from the previously learned sequence (i.e., no WM salience), no OPE, but a SNARC effect was found (Ginsburg et al., 2014). In these conditions, participants memorised the numbers for a later recall and maintained them, whereas retrieval of the numbers during the direct or indirect classification tasks was not required. Interestingly, even the SNARC effect reflecting the spatialization of LTM ordinality or cardinality might depend on the sequence maintained in WM, although the sequence was irrelevant for the task: In the respond-to-all magnitude classification, a SNARC effect was only observed for the numbers that were part of the memorised sequence but not for other numbers. In line with the findings by Ginsburg et al. (2014), van Dijck et al. (2014) found an OPE for numbers in a Posner cueing task with (Experiment 2) but not without retrieval of the memorised sequence (Experiment 1). The OPE in the Posner cueing task with retrieval was replicated for letters (Experiment 3). Thus, the activation of the memorised sequence in WM by its retrieval seems to be a prerequisite for the OPE.

Storing a sequence in WM was only a secondary task in the experiments by van Dijck and Fias (2011) and Ginsburg et al. (2014). Nevertheless, the salience of WM was high in the judgement task because of the go/no-go design, which might have triggered an OPE. The literature overview in Table C1 (online Supplementary Material C) shows that the WM salience can be evaluated as high in all go/no-go designs that have so far been used. In this regard, the current study is unique, as it combines the instruction to store an ordered sequence in WM with a go/no-go design, while achieving different levels of WM salience, as described further below.

## Spatial mapping of non-numerical ordered stimuli

In its original form, the WM account excluded the coexistence of the SNARC effect and the OPE; the SNARC effect should disappear or be reduced if a deviating order should be remembered in WM. However, as opposed to the findings by van Dijck and Fias (2011) and by Ginsburg et al. (2014), the coexistence of the SNARC and the OPE has been observed in later studies by Ginsburg and Gevers (2015) and by Huber et al. (2016). Moreover, ordinality has been demonstrated to influence spatial response preferences for numbers, even in cases where no ordinal number sequence is learned prior to the task. This was illustrated by Koch et al. (2023). In a parity judgement setup, they disentangled the inherent ordinality of numbers from their magnitude by using number sets such as 1, 2, 3, and 8 and testing whether ordinality or magnitude determines the pattern of response times (RT) better. Typically, the SNARC effect and the OPE are quantified by the slopes obtained by regressing, for each participant separately, the dRTs (i.e., the difference in the average RT between right- and left-hand responses for each magnitude or position) to the magnitudes or positions. In this example, there are five magnitude units between 3 and 8, but these are the third and fourth numbers when ordering the set by increasing magnitude so that there is only one ordinality unit between 3 and 8. Koch et al. (2023) investigated whether both numbers or positions explained a unique proportion of variance in dRTs, and found influences by both inherent ordinality and magnitude for the spatialization of numbers. Ginsburg and Gevers (2015) assume that the SNARC effect and the OPE activate different representations and can therefore coexist, further suggesting that the MNL and the WM account are not mutually exclusive. More precisely, the SNARC effect reflects the spatial representation of the canonical order of numbers being stored in LTM, whereas the OPE reflects the spatial representation of either an overlearned order in LTM (e.g., the alphabet in the case of letters) or a newly learned order being stored in WM. Abrahamse et al. (2016) claim that a template for a spatial mental representation is most likely stored in LTM, but the template is then retrieved from LTM and used in WM. According to their view, the items under question are filled into this template only in WM, no matter whether a sequence is already familiar (i.e., stored in LTM and retrieved to WM) or only newly learned (i.e., only stored in WM, but not in LTM). In contrast, Guida and Campitelli (2019) claim in their expertise account that spatial information can be stored together with orders in LTM and that the association between orders and space is not always built in WM. Specifically, in their view, spatialization already happens in LTM for canonical orders (such as of numbers or letters), and only takes place in WM for newly learned orders, for which no spatial information exists in LTM. It is not clear, however, how this account can explain the disappearance of the SNARC effect when WM resources are depleted (e.g., Ginsburg et al., 2014; van Dijck et al., 2009). The simultaneous observation of the SNARC effect and the OPE led to the development of a new hybrid explanation: Not a single underlying representation determines spatial-numerical associations, but instead an interplay of two or more components, such as LTM representations and WM contents (van Dijck et al., 2015). In line with this, differential effects of anodal tDCS (transcranial Direct Current Stimulation) have been found for different stimulus types (i.e., number words, weekdays, and months), suggesting that multiple codes (e.g., verbal markedness, spatial verbal instruction, sequential WM, and visuospatial stimulation) are responsible for spatial associations of ordinal stimuli (Schroeder et al., 2017b).

As mentioned earlier, previous research has been able to show spatial associations not only for numbers but also for non-numerical stimuli. The current study aimed at answering the question of whether the WM account also holds for other ordered stimuli that do not convey any magnitude information (i.e., metathetic dimensions, for which ATOM is not claimed to hold), such as letters. Similar to numbers, letters have a fixed inherent order (i.e., we count “one, two, three,...,” and we recite the alphabet “A, B, C,...”). If we compare numbers with letters or other familiar sequences such as the alphabet, numbers are special. The order of numbers is based on their cardinality (e.g., the number 3 corresponds to three elements and the number 4 to four elements), and they have a quantitative meaning (e.g., 4 is larger than 3). This is not the case for letters (e.g., B is not more or less than A): While numbers convey both magnitude and ordinality, letters possess only ordinality (e.g., B comes after A in the alphabet) without magnitude. As shown by Zorzi et al. (2006) in mental interval bisection tasks, left-spatial-neglect patients’ performance was influenced by the interval length for number stimuli (i.e., bias to the left in small intervals like 1–3, but bias to the right in large intervals like 1–9) but not for letter stimuli (e.g., no evidence for a systematic performance difference between L–N and L–T). The authors thus concluded that the mental representations of letters and numbers are qualitatively different. In conclusion, it remains uncertain what spatial mental representations of non-numerical metrics, characterised by ordinal but lacking cardinal information, entail and whether they are governed by shared processes with spatial associations of numbers.

## The current study and hypotheses

To find out whether spatial associations for ordinal sequences depend on the salience of WM demands, we investigated letter-space associations while manipulating the degree to which WM is involved in the task. Crucially, by using letters, we were able to disentangle ordinality from magnitude in three experiments. We adapted the standard WM paradigm of van Dijck and Fias (2011), taking into account findings by Ginsburg et al. (2014) that the retrieval of the memorised sequence during the classification task is essential to observe an OPE. Experiment 1 did not include any encoding phase (i.e., no WM salience) and served as manipulation check to see whether a task set up with a go/no-go design only influences spatial associations (i.e., *control condition*). In two further experiments, we manipulated the need to retrieve the memorised letter sequence during the classification task by using different “no-go” stimuli. In Experiment 2, we used non-letter symbols like ‡, △, ≡, and ∇ as “no-go” stimuli, which could easily be identified as a “no-go” trial. The memorised sequence had to be maintained during the classification task but needed to be recalled only later (i.e., *maintenance condition*), creating intermediate WM salience. In Experiment 3, we used letters that were not part of the letter sequence memorised in the encoding phase (i.e., *retrieval condition*) as “no-go” trials. Thus, it was necessary to retrieve the memorised sequence to identify a letter as a “go” trial (i.e., when it was part of the memorised sequence) or as a “no-go” trial (i.e., when it was not part of the memorised sequence), creating high WM salience.

According to Ginsburg et al. (2014), an OPE is only elicited if the memorised ordinal sequence needs to be retrieved from WM during the judgement task. In contrast, only spatial associations based on LTM can be observed if the memorised sequence is merely maintained in WM without being retrieved. Hence, if the same mechanisms apply to letters as to numbers, no OPE but a SAARC effect should be found in the maintenance condition (Experiment 2). At the same time, an OPE but no SAARC effect should be found in the retrieval condition (Experiment 3).

The present study was preregistered (https://osf.io/945uq), and all datasets are publicly available on the Open Science Framework, together with the corresponding R scripts for the analysis (https://osf.io/v84m5/). Note that in the preregistration, we wrote that we wanted to compare the strength of both effects with one another; however, upon closer inspection, we realised that it is not the difference between the two effects that is meaningful for the underlying mechanism. Instead, what matters is solely the presence of an OPE as a marker for WM involvement or of a SAARC as a marker for LTM involvement. We therefore adapted our hypotheses as described above and our analyses as described below.

## General method

### Overview and design

In three online experiments with a between-subjects design, which differed in the salience of the WM instruction, we used a go/no-go paradigm that closely followed the study by van Dijck and Fias (2011) and subsequent studies (Ginsburg et al., 2014; Ginsburg & Gevers, 2015). The main differences were (i) that stimuli were letters instead of numbers to rule out cardinality and (ii) that the used indirect task was a consonant-vowel classification instead of a parity judgement. Furthermore, (iii) each participant had to remember only one sequence across the entire experiment in the initial encoding phase. In contrast, previous studies consisted of several experimental blocks each including (1) an encoding phase, (2) a classification phase, and (3) a control phase, such that there was one new sequence per block (number of blocks per study: 20 respond-to-all or 20 go/no-go in Ginsburg et al., 2014; 20 respond-to-all mixed with 20 go/no-go in Ginsburg & Gevers, 2015; 54 go/no-go in Huber et al., 2016; 20 go/no-go in van Dijck and Fias, 2011). Importantly, the salience of WM demands was systematically increased from Experiment 1 (no WM salience: no ordinal sequence maintained) over Experiment 2 (intermediate WM salience: ordinal sequence maintained and retrieved after the task) to Experiment 3 (high WM salience: ordinal sequence maintained and retrieved during the task), as illustrated in Table 1.

**Table 1.** Scheme of the experimental procedure with ascending salience of WM demands from Experiment 1 to Experiment 3. Consonant-vowel classification was used in all three experiments. Experiments 1 and 2 only differed regarding the encoding and control phases, which were absent in Experiment 1 (i.e., without any WM task) and present in Experiments 2 and 3 (i.e., including a WM task). Experiments 2 and 3 only differed regarding the “no-go” stimuli, which were symbols in Experiments 1 and 2 (i.e., no retrieval from WM required for the distinction between “go” and “no-go” stimuli) and letters in Experiment 3 (i.e., retrieval from WM required).

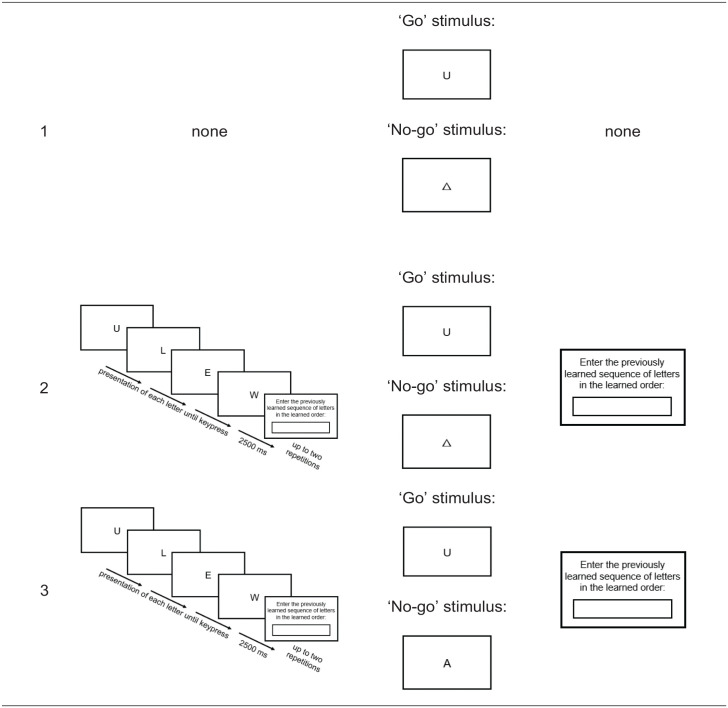

### Sample size

We conducted an a priori sample-size determination analysis in JAMOVI (The Jamovi Project, 2023) to determine sample size (see our preregistration: https://osf.io/945uq). To obtain a power of 0.8 for detecting the minimally relevant effect size of Cohen’s *d* = 0.25 with the Bonferroni-corrected significance level of α = .05 / 3 = .017 in one-tailed independent-samples *t-*tests to compare the SAARC effect and the OPE between experiments, a sample size of 282 participants per experiment (thus a total *N* = 846) would have been required. Between July and December 2021, the target sample size was not achieved, despite intensive recruitment efforts (via round mails at the University of Tübingen, Facebook, Instagram, Twitter, Reddit, and personal acquaintances), and despite adding an English version, which might be due to the COVID-19 pandemic. We stopped data collection with a total of 515 participations (German version: 474, English version: 41).

Importantly, instead of running three independent-samples *t*-tests as stated in the preregistration, we ran five one-sample *t*-tests, as they fit our research questions better. Therefore, the preregistered sample-size determination analysis was invalid for the analyses that we eventually decided to apply, and we conducted a power-determination analysis (as described by Giner-Sorolla et al., 2024, see R scripts at https://osf.io/v84m5/). The reached sample sizes prior to exclusions due to data preprocessing were 178, 169, and 168 for Experiments 1, 2, and 3, respectively; 149, 105, and 107 datasets remained for the analysis after preprocessing. The determined power levels for detecting the minimally relevant effect size of Cohen’s *d* = 0.25 with the Bonferroni-corrected significance level of α = .05 / 5 = .01, and the size of these final samples were poor, namely 0.43, 0.30, and 0.31. In a subsequent effect-size sensitivity analysis (as described by Giner-Sorolla et al., 2024), we found out that with a power level of 0.80, the applied statistical tests would be able to detect true underlying effects with *d* = 0.37, *d* = 0.44, and *d* = 0.44 with the final sample sizes. Note that the final samples are still more than four times larger than in the original studies by van Dijck and Fias (2011) and Ginsburg et al. (2014) on which our work is based and who tested between 21 and 39 participants per experiment, and that *d* = 0.4 is reasonable as the smallest effect size of interest in psychology according to Brysbaert (2019). All participants in the present study were adults with a minimum age of 18 years. As compensation, participants could either receive course credits or participate in a lottery, with the opportunity to win a voucher worth €50, with odds of one in seven.

### Materials and stimuli

The three online experiments were programmed in jsPsych (de Leeuw, 2015). We hosted the experiments on SoSci Survey (Leiner, 2021), to assign participants randomly to the experiments and obtain their email addresses for the lottery, and on Pavlovia (Pavlovia Surveys (Open Science Tools), 2021), to run the experiment with the tasks and questionnaires on demographic and control variables.

**Figure 1.**
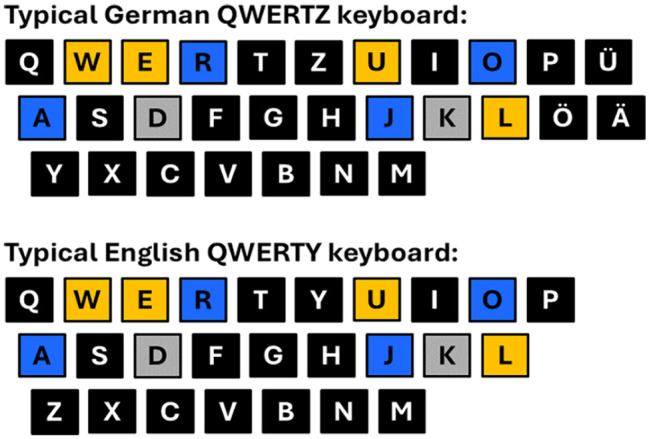
Letter positions on a typical German QWERTZ keyboard (upper panel) and on a typical English QWERTY keyboard (lower panel), with yellow/blue colour-coding for the used letter sets E, L, U, W and A, J, O, R, respectively, and with the response keys coloured in grey.

To be able to compare results across the three experiments, we used the same stimulus sets in each experiment, with two sets being put together of the same four letters whose order was changed (see Table 2).^1^ The letter sets were composed of two vowels and two consonants, with one vowel and one consonant on the left and right sides of the keyboard, respectively (see Figure 1). In addition, the letters were selected to cover a wide range of the alphabet, to be distributed as evenly as possible over the alphabet, and to not form words or familiar abbreviations (e.g., company names). The resulting letter sequences were [O J R A], [R A O J], [L E W U], and [U L E W] (see the online Supplementary Material A for a detailed description of the letter selection procedure).

**Table 2.** Overview of the memorised letter sequences as well as “go” and “no-go” stimulus sets, separately for each experiment, with each row corresponding to one between-subjects condition participants were randomly assigned to (note that within each of conditions, participants were randomly assigned to one of two response-to-key assignment orders: Participants either responded to vowels with D and to consonants with K in the first half and to consonants with D and to vowels with K in the second half, or vice versa).

Experiment	(1) encoding phase, (3) control phase	(2) classification phase	
	Memorised sequence	‘Go’ stimulus set	‘No-go’ stimulus set
Exp. 1	none	A, J, O, R	‡, △, ≡, ∇
	none	E, L, U, W	‡, △, ≡, ∇
Exp. 2	[O J R A]	A, J, O, R	‡, △, ≡, ∇
	[R A O J]	A, J, O, R	‡, △, ≡, ∇
	[L E W U]	E, L, U, W	‡, △, ≡, ∇
	[U L E W]	E, L, U, W	‡, △, ≡, ∇
Exp. 3	[O J R A]	A, J, O, R	E, L, U, W
	[R A O J]	A, J, O, R	E, L, U, W
	[L E W U]	E, L, U, W	A, J, O, R
	[U L E W]	E, L, U, W	A, J, O, R

Each participant was assigned to only one stimulus set (out of two in Experiment 1, where the order of the letters did not matter, and out of four in Experiments 2 and 3, where the order of the letters did matter), which were counterbalanced between participants. The “go” stimuli in the classification phase consist of four different letters (in Experiments 2 and 3, the “go” stimuli were the four letters learned in the encoding phase). The “no-go” stimuli differed between the experiments: In Experiments 1 and 2, we used the symbols ‡, △, ≡, and ∇. In Experiment 3, we used the letters of the respective other stimulus set as “no-go” stimuli (e.g., when the stimulus set was [O J R A] or [R A O J], the letters E, L, U, and W were used as “no-go” stimuli, see Table 2). Importantly, the used letters did not differ between experiments (see Table 2), so that differences between experiments cannot be attributed to stimuli used in the consonant-vowel classification task.

### Procedure

This study was approved by the Ethics Committee of the University of Tübingen’s Department of Psychology. In the beginning, participants were informed about the study, eligibility requirements (i.e., participation with a desktop, laptop, or netbook), and data protection. After their informed consent, they were randomly assigned to one of the three experiments, and subsequently to one of two “go” stimulus sets (in Experiment 1) or to one of four-letter sequences to be learned (in Experiments 2 and 3), as summarised in Table 2.

*1. Encoding phase*: In Experiments 2 and 3, participants were instructed to memorise a sequence consisting of four letters. The letters were presented one after another in the centre of the screen. Participants could move from one letter to the next via keypress so that they determined the presentation speed. After 2500 ms, participants should type the letter sequence. In case of a mistake, the letter sequence was presented again, with a maximum of three attempts.
*2. Classification phase*: In the main part of all three experiments, participants were instructed to decide via key press (D or K) whether a presented letter was a consonant or a vowel. In one half of the experiment, participants were instructed to press the left response key (D) when they saw a consonant and the right response key (K) when they saw a vowel. In the other half, the response-to-key assignment was reversed. Response-to-key assignment order was counterbalanced between participants. In Experiment 1, participants were instructed to respond to letters (“go” stimuli) but not to symbols (“no-go” stimuli, no WM salience). Participants were instructed to respond only to memorised letters (“go” stimuli) but not to symbols (“no-go” stimuli) in Experiment 2 (intermediate WM salience) or to letters that were not part of the learned letter sequence (“no-go” stimuli) in Experiment 3 (high WM salience). Each trial started with the presentation of a fixation cross for 500 ms, followed by the presentation of the stimulus until a response key was pressed or after a maximum of 1500 ms. During each inter-trial interval, a blank screen was presented for 100 ms. Each stimulus was repeated 20 times per response-to-key assignment, resulting in a total of 320 trials (8 stimuli x 2 assignments x 20 repetitions), that is 160 “go” trials and 160 “no-go” trials. The order of stimulus presentation was pseudorandomized, so that the same stimulus was never repeated in consecutive trials. After every 50 trials, there was an opportunity to take a short break. At the beginning of the respective experiment and after changing the response-to-key assignment, 16 practice trials were presented, with each stimulus (four “go” stimuli and four “no-go” stimuli) being presented twice. In practice trials, accuracy feedback was displayed for 1000 ms. If fewer than 75% of the stimuli were answered correctly, the practice trials were repeated.
*3. Control phase*: In Experiments 2 and 3, participants should type the letter sequence learned beforehand in the encoding phase (open response format). As in the encoding phase, the letters could be entered up to three times.

After these phases, a questionnaire was presented, asking participants about:

- memory strategy (open response format, e.g., whether they had written down the letter sequence to be memorised)
- age and gender (*male*, *female*, *diverse*)
- mother tongue and language skills (last grade: 0—*very poor* to 10—*very good*; diagnosed reading/spelling disability or dyslexia: *yes*, *no*)
- knowledge of a language with a reading/writing direction from right to left (*yes*, *no*)
- handedness (*right-handed*, *left-handed*, or *ambidextrous*), starting hand when counting with fingers (*left*, *right*), and stability of this starting hand (*always*, *usually*, *sometimes*)
- alphabetical knowledge (RT and correctness of the answer to four questions about the position of the letters in the alphabet: *Does the letter “K” come before “M” in the alphabet? Does the letter “T” come before “L” in the alphabet? Does the letter “R” come before “O” in the alphabet? Does the letter “J” come before “Q” in the alphabet?*)
- data quality (how noisy the environment was during participation: *extremely quiet*, *very quiet*, *a little quiet*, *a little noisy*, *very noisy*, *extremely noisy*; whether participants would use their data if they were the experimenter: *yes*, *not all*, *no*)

### Data preprocessing

All data preprocessing steps, statistical power considerations, and data analyses were run in the statistical computing software R (R Core Team, 2023), using the R packages *clinfun* (Seshan & Whiting, 2023), *dplyr* (Wickham, François, et al., 2023), *ggplot2* (Wickham et al., 2024), *ggpubr* (Kassambara, 2023), *plyr* (Wickham, 2023a), *pwr* (Champely et al., 2020), *stringr* (Wickham, 2023b) *tidyr* (Wickham, Vaughan, et al., 2023), and *tidyverse* (Wickham, 2023c). The analysis R scripts as well as the anonymized data are available on the Open Science Framework at https://osf.io/v84m5/.

Our analysis was based on RT data (i.e., time from the stimulus onset until a key press) of the correct responses in the “go” trials. Exclusion of outliers and filtering of the data were performed in line with the preregistration (https://osf.io/945uq) and analogous to the procedure of Cipora et al. (2019). Specifically, we excluded entire datasets from:

- incomplete participations (i.e., participants quit before the end)
- participants who used other devices than a desktop, laptop, or netbook
- participants who performed the task in a very or extremely noisy environment
- participants who declared that they would not use their data or not all of it if they were the experimenter
- participants who correctly refrained from responding in fewer than 80% of all “no-go” trials
- participants who could not reproduce the learned letter sequence correctly at their first try in the control phases in Experiments 2 or 3 (note that we did not specify in the preregistration whether participants who could reproduce the sequence correctly at their second or third try would also be excluded, but since memorising the exact letter sequence correctly is a prerequisite for measuring the OPE, we decided to be strict here)
- participants who indicated that their memory strategy had been to write down the letter sequence to be memorised in the encoding phase (note that this criterion was not preregistered, but having the correct letter sequence in WM rather than using external aids is crucial for the OPE)

Afterwards, we removed all incorrectly responded trials and all trials with RTs below 250 ms (as it was done by Cipora et al., 2019 and van Dijck & Fias, 2011). Then, as preregistered, a sequential trimming procedure was conducted within each participant, sequentially removing all RTs beyond ± 3 *SD* from the individual mean RT. Finally, full datasets of participants with fewer than 70% of valid trials left per letter in each response-to-key assignment were excluded.

### Data analysis

The data analysis was based on the repeated-measures regression procedure proposed by Lorch and Myers (1990) and first applied to the SNARC effect by Fias et al. (1996). Accordingly, we first calculated mean RTs for each participant and each letter separately for both response sides. Then, mean RT differences (dRTs) were computed for each letter by subtracting mean RTs given with the left hand from mean RTs given with the right hand. Negative dRTs indicate faster responses with the right hand, while positive dRTs indicate faster responses with the left hand. Subsequently, linear regressions were performed for each participant *i*, with either (1) the position of the letters in the alphabet (e.g., 15, 10, 18, 1 for set [O J R A]) or (2) the position of the letters within the memorised letter sequence (i.e., 1, 2, 3, 4) as the predictor variable and dRTs as outcome variable:

(1) SAARC: *dRT_i_* *=* β_0_ *+* β_1_ * position_(alphabet)i_ *+* *ε_i_*
(2) OPE: *dRT_i_* *=* β_2_ *+* β_3_ * position_(sequence)i_ *+* *ε_i_*

Both regression models were fit for participants in Experiments 2 and 3, whereas only the Model (1) was fit for participants in Experiment 1, as no ordinal letter sequence was learned in this control condition. The unstandardized regression slopes β_1_ and β_3_ indicate the change in right- over left-hand advantage in milliseconds per increase of one position unit. The unstandardized slopes β_1_ resulting from Model (1) indicate the SAARC effect, while the unstandardized slopes β_3_ from Model (2) indicate the OPE. We standardised and Fisher-z-transformed the regression slopes within each experiment, such that they can be interpreted as *effect sizes*. The main dependent variable was standardised slopes, which are unaffected by individual RTs and individual variance in RTs. The presence of a SAARC effect and an OPE at the group level was tested by comparing these slopes to zero using a one-tailed, one-sample *t*-test (as in previous literature, e.g., Ginsburg et al., 2014; Ginsburg & Gevers, 2015; van Dijck & Fias, 2011). We accounted for multiple hypothesis tests via the Bonferroni-Holm correction,^2^ starting with a significance level of α = .05 / 5 = .01 for the test with the lowest corresponding *p* value. Note that we preregistered to run three tests because we originally planned to compare the SAARC effect and the OPE within each experiment. However, because quantitatively comparing slopes for two qualitatively different effects is theoretically not meaningful, we decided to test the SAARC effect and the OPE separately within each experiment, resulting in five tests. For data exclusions at the participant level and for a summary of the main results for all three experiments, see Tables 3 and 4, respectively.

**Table 3.** Data exclusions at the participant level for each experiment.

Exclusion criterion	Exp. 1	Exp. 2	Exp. 3
No serious participation	0	2	1
Other device than desktop, laptop, netbook	0	0	0
Incomplete participation	3	2	3
Noisy environment	2	3	0
Would not use (all) data as experimenter^a^	15	24	16
Fewer than 80% valid ‘no-go’ trials	0	0	5
Fewer than 70% valid ‘go’ trials per cell	13	4	9
Incorrect recall of letter sequence at 1st try	-	34	37
Correct recall at 2nd try / 3rd try	-	10 / 7	19 / 5
Incorrect recall even at 3rd try / noted down		17 / 9	13 / 8

a Regarding the answers to the question whether participants would use their data if they were the experimenter, across all three experiments, the average accuracy (i.e., proportion of correctly responded “go” trials and correctly non-responded “no-go” trials among all trials) was 0.948 for *yes*, 0.893 for *not all*, and 0.813 *no*, suggesting an accurate self-assessment of by the participants for their own performance relative to others.

**Table 4.** SAARC and OPE regression slopes resulting from the three experiments, with asterisks indicating significant results (i.e., the respective slope being significantly different from zero using the Bonferroni-Holm corrected significance level).

Experiment	SAARC effect		OPE	
	slope	*p*	slope	*p*
Exp. 1 (control condition)	−0.42	< .001*	-	-
Exp. 2 (maintenance condition)	−0.40	< .001*	−0.08	.142
Exp. 3 (retrieval condition)	−0.28	< .001*	−0.27	< .001*

## Experiment 1

Gevers et al. (2003) showed spatial response preferences for letters based on their ordinal position in the alphabet: Letters at the beginning of the alphabet were responded to faster with the left than with the right hand, while the reverse response pattern was observed for letters at the end of the alphabet (see their Experiment 2, order-irrelevant task). In Experiment 1, in addition to letters as “go” stimuli, symbols were presented as “no-go” stimuli. This control condition served as a manipulation check and makes it possible to integrate our findings into the existing literature on response preferences for letters, as this is the first study on letter-space associations using a go/no-go design. We expected faster left-hand responses for letters at the beginning of the alphabet and faster right-hand responses for letters at the end of the alphabet (i.e., a SAARC effect), replicating the results by Gevers et al. (2003). As no letter sequence had to be learned in this experiment, WM was not salient and no OPE could be computed for this condition.

### Method

#### Participants

In all, 178 individuals participated in this online experiment. After exclusions (see Table 3), datasets from 149 participants (German version: 136, English version: 13) remained for the analysis. The average age of these participants was 26.75 years (*SD* = 10.20, range: 18 to 72). Ninety-nine participants identified as female and 50 as male; 130 participants reported being right-handed, 16 left-handed, and three ambidextrous; and 130 participants indicated German as their native language (other native languages were English, Chinese, Turkish, Spanish, Bengali, Portuguese, Taiwanese, Hungarian, French, and Hindi; none of them with reading/writing direction from right to left).

#### Stimuli, design, and procedure

Participants completed a consonant-vowel classification task in which “no-go” stimuli were ‡, △, ≡, and ∇. They were assigned to one of four conditions in a 2 (“go” stimuli: A, J, O, R vs. E, L, U, W) × 2 (response-to-key order: start with vowel-left and consonant-right vs. start with vowel-right and consonant-left) design, as illustrated in Table 2.

### Results

After applying exclusion criteria at the participant level (except for two: minimum of 80% valid “no-go” trials and minimum of 70% valid “go” trials per cell), 6.96% of the trials were excluded from the analysis (first 5.53% due to incorrect responses, another 0.02% because of anticipations with RTs below 250 ms, and another 1.41% within the sequential trimming procedure). The mean RT was 584.7 ms (*SD* = 134.5 ms). Mean RTs per letter and mean accuracies per letter set can be found in the online Supplementary Material B (Tables B1 and B2).

**Figure 2.**
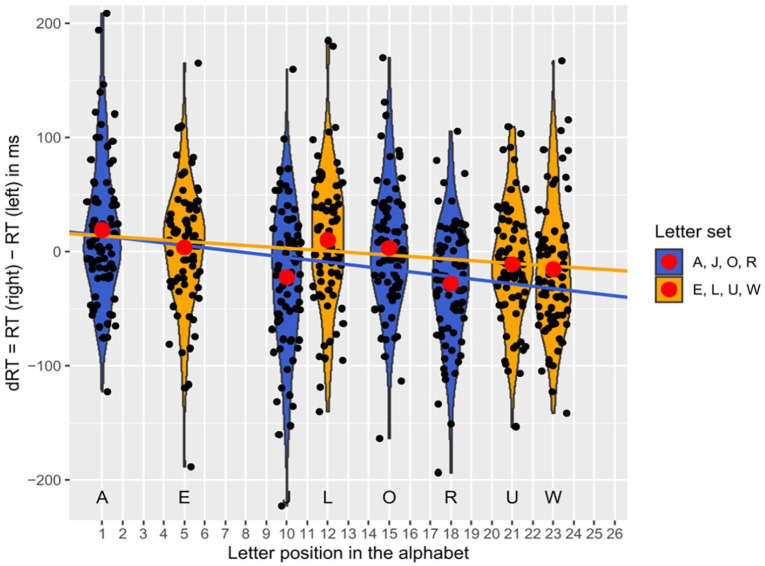
Differences in reaction times (dRT) with the right and left hand depending on the position of the letter in the alphabet (SAARC effect) in Experiment 1 (manipulation check), with black dots being jittered mean dRTs per participant for the respective position surrounded by violins, red dots being mean dRTs averaged over participants, and regression lines reflecting the SAARC slopes averaged over participants (between-subjects assignments are colour-coded in yellow/blue for the letter sets E, L, U, W and A, J, O, R, respectively).

The regression analysis revealed a significant negative standardised slope for the letter position in the alphabet, β_1_ = −0.42, *t*(148) = −5.54, *p* < .001 (using α = .0125). Thus, as expected, a significant SAARC effect was found, indicating an increase of right-hand advantage with later positions of the letter in the alphabet, see Figure 2.

### Discussion

These results are a replication of Gevers et al.’s (2003) and the observed SAARC effect confirms the spatial response preferences for letters according to their order in the alphabet (i.e., according to their ordinal representation in LTM). To conclude, the consonant-vowel classification task including non-letter “no-go” trials can be used to measure spatial associations.

## Experiment 2

According to Ginsburg et al. (2014), in the case of numerical stimuli, the observation of the OPE depends on the retrieval of the learned sequence. If the sequence was only maintained for later retrieval, no OPE was found; instead, a regular SNARC effect emerged. In this experiment, we investigate whether this pattern can also be shown for non-numeric ordinal sequences like letters. Analogous to the results of Ginsburg et al. (2014), we assume that, if the learned sequence was merely to be maintained in WM for later recall, and the WM salience in the judgement task was thus at best intermediate, no OPE but a SAARC effect should be found.

### Method

#### Participants

The sample consisted of 169 participants. After exclusions (see Table 3), datasets of 105 participants (German version: 97, English version: 8) with an average age of 25.79 years (*SD* = 10.71, range: 18 to 71) were analysed. Of these participants, 71 identified as female, 33 as male, and one as non-binary. 98 participants reported to be right-handed, five to be left-handed, and two to be ambidextrous. The native language was German for 90 participants (other native languages were English, German, Luxembourgish, Turkish, Russian, Chinese, Arabic, Italian, Greek, Hindi, and Spanish). Only one participant reported a native language with reading/writing directions from right to left.^3^

#### Stimuli, design, and procedure

As in Experiment 1, participants completed a consonant-vowel classification task in which “no-go” stimuli were ‡, △, ≡, and ∇. They were assigned to one of eight conditions in a 4 (“go” stimuli and letter sequence: [O J R A] vs. [R A O J] vs. [L E W U] vs. [U L E W]) × 2 (response-to-key order: start with vowel-left and consonant-right vs. start with vowel-right and consonant-left) design, as illustrated in Table 2.

### Results

After applying exclusion criteria at the participant level (except for two: minimum of 80% valid “no-go” trials and minimum of 70% valid “go” trials per cell), 5.79% of the trials were excluded from the analysis (first 4.24% due to incorrect responses, none because of anticipations with RTs below 250 ms, but another 1.55% within the sequential trimming procedure). The mean RT in the classification task was 563.5 ms (*SD* = 124.5 ms). Mean RTs per letter and mean accuracies per letter set can be found in the online Supplementary Material B (Tables B1 and B2).

**Figure 3.**
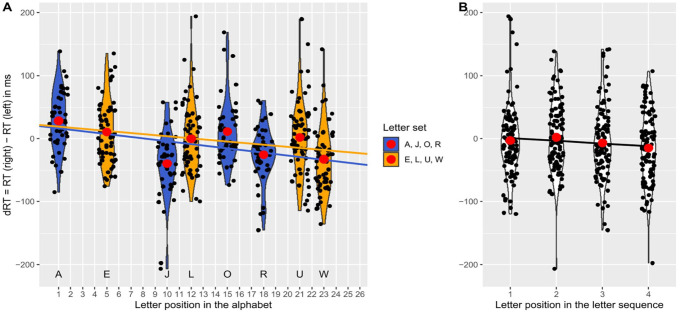
Differences in reaction times (dRT) with the right and left hand depending on the position of the letter in the alphabet (SAARC effect, Panel A) or in the memorised sequence (OPE, Panel B) in Experiment 2 (maintenance condition), with black dots being jittered mean dRTs per participant for the respective position surrounded by violins, red dots being mean dRTs averaged over participants, and regression lines reflecting the SAARC slopes averaged over participants in Panel A (between-subjects assignments are colour-coded in yellow/blue for the letter sets E, L, U, W and A, J, O, R, respectively) and the OPE slopes in Panel B.

The first regression analysis revealed a significant negative standardised slope for the letter position in the alphabet, β_1_ = −0.40, *t*(104) = −6.00, *p* < .001 (using α = .0100). Thus, as expected, and as in Experiment 1, a significant SAARC effect was found, indicating an increase of right-hand advantage with later positions of the letter in the alphabet (see Figure 3, Panel A). In contrast, the second regression analysis did not reveal a significant negative standardised slope for the letter position in the learned sequence, β_3_ = −0.08, *t*(104) = −1.08, *p* = .142 (using α = .0500). Thus, as expected, no significant OPE was found (see Figure 3, Panel B).

### Discussion

Our results extend the results of Ginsburg et al. (2014) to non-numerical ordinal stimuli like letters. Like Ginsburg et al. (2014), we found no OPE when the memorised sequence was merely maintained for a later recall. Instead, in line with their finding of a SNARC effect, we observed a SAARC effect (i.e., a spatial mapping according to the ordinal representation of letters in LTM).

## Experiment 3

As explained above, Ginsburg et al. (2014) found that the OPE for numerical stimuli depends on the retrieval of the learned sequence during the classification task. To investigate whether this is also true for non-numerical ordinal stimuli like letters, we used letters deviating from the memorised letter sequence as “no-go” stimuli during a consonant-vowel classification task. Using other letters as “no-go” stimuli requires participants to retrieve the memorised letter set during the classification task itself (i.e., retrieval condition), creating high WM salience. We expected the same mechanism to apply to letters as to numbers, such that an OPE but no SAARC effect would be found in this experiment.

### Method

#### Participants

In all, 168 individuals participated in this experiment. After exclusions (see Table 3), the final sample for the data analysis consisted of 107 participants (German version: 100, English version: 7) with an average age of 25.43 years (*SD* = 7.31, range: 18 to 64). 75 of them identified as female, 30 as male, and two as non-binary. Furthermore, 96 participants indicated being right-handed, nine left-handed, and two ambidextrous. 96 participants reported German as their native language (other reported native languages were English, Turkish, Kurdish, Bulgarian, Polish, Greek, Bosnian, Spanish, Ukrainian, Persian, and Hindi). Only one participant indicated a native language with reading and writing directions from right to left.^4^

#### Stimuli, design, and procedure

As opposed to the previous two experiments, the “no-go” stimuli were letters instead of symbols. Participants were assigned to one of eight conditions in a 4 (“go” stimuli and letter sequence: [O J R A] vs. [R A O J] vs. [L E W U] vs. [U L E W]) × 2 (response-to-key order: start with vowel-left and consonant-right vs. start with vowel-right and consonant-left) design, as illustrated in Table 2.

### Results

After applying exclusion criteria at the participant level (except for two: minimum of 80% valid “no-go” trials and a minimum of 70% valid “go” trials per cell), 6.44% of the trials were excluded from the analysis (first 5.04% due to incorrect responses, none% because of anticipations with RTs below 250 ms, but another 1.40% within the sequential trimming procedure). The mean RT in the classification task was 615.3 ms (*SD* = 150.0 ms). Mean RTs per letter and mean accuracies per letter set can be found in the online Supplementary Material B (Tables B1 and B2).

**Figure 4.**
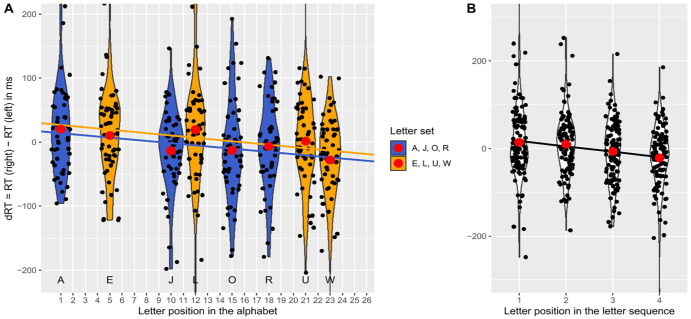
Differences in reaction times (dRT) with the right and left hand depending on the position of the letter in the alphabet (SAARC effect, Panel A) or in the memorised sequence (OPE, Panel B) in Experiment 3 (retrieval condition), with black dots being jittered mean dRTs per participant for the respective position surrounded by violins, red dots being mean dRTs averaged over participants, and regression lines reflecting the SAARC slopes averaged over participants in Panel A (between-subjects assignments are colour-coded in yellow/blue for the letter sets E, L, U, W and A, J, O, R, respectively) and the OPE slopes in Panel B.

The first regression analysis revealed a significant negative standardised slope for the letter position in the alphabet, β_1_ = −0.28, *t*(106) = −3.91, *p* < .001 (using α = .0167). Thus, as expected, and as in Experiments 1 and 2, a significant SAARC effect was found, indicating an increase of right-hand advantage with later positions of the letter in the alphabet (see Figure 4, Panel A). Moreover, the second regression analysis revealed a significant negative standardised slope for the letter position in the learned sequence, β_3_ = −0.27, *t*(106) = −3.51, *p* < .001 (using α = .0250). Thus, the expected OPE was found (see Figure 4, Panel B).

### Discussion

In line with Ginsburg et al. (2014), we found an OPE in this experiment, in which retrieval of the memorised set was required during the classification task (retrieval condition). However, we also found a SAARC effect. Thus, the spatial mapping of letters seems to occur both according to their ordinal sequence in WM (i.e., learned letter sequence) and in LTM (i.e., alphabetical sequence).

## Joint exploratory analyses

### Comparison of the SAARC effect between letter sets

**Figure 5.**
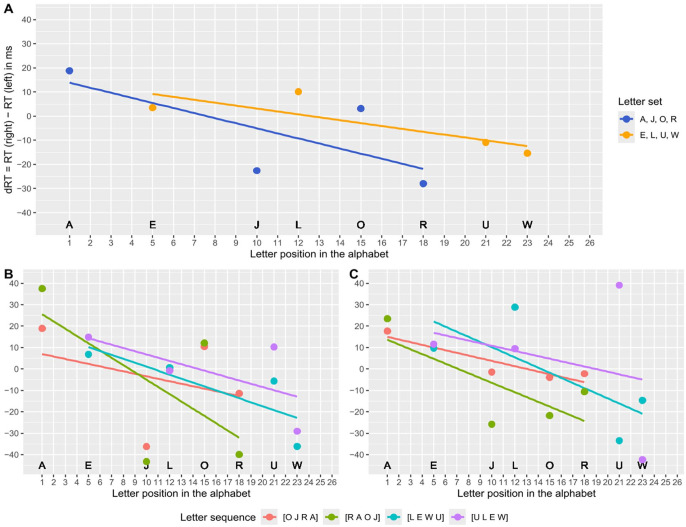
Differences in reaction times (dRT) with the right and left hand depending on the position of the letter in the alphabet (SAARC effect) and averaged over participants in Experiment 1 (control condition, Panel A), Experiment 2 (maintenance condition with intermediate WM salience, Panel B), and Experiment 3 (retrieval condition with high WM salience, Panel C), for each letter set separately.

To examine the SAARC effect in more detail, we also calculated the regressions per letter set (Experiment 1) or learned letter sequence (Experiments 2 and 3) separately. Figure 5 illustrates the resulting average dRTs per letter position in the alphabet and the SAARC slopes for each experiment. All unstandardized and standardised SAARC slopes are descriptively negative. In Experiment 1, the standardised SAARC slopes did not differ significantly between A, J, O, R (β_1_ = −0.40, *n* = 75) and E, L, U, W (β_1_ = −0.44, *n* = 74), when comparing with a two-sided independent-samples *t*-test, *t*(147) = 0.27, *p* = .789. Moreover, in Experiment 2, the standardised SAARC slopes did not differ significantly between the four memorised letter sequences [O J R A] (β_1_ = −0.37, *n* = 22), [R A O J] (β_1_ = −0.61, *n* = 22), [L E W U] (β_1_ = −0.36, *n* = 32), and [U L E W] (β_1_ = −0.31, *n* = 29), when testing the main effect of the between-subject factor letter sequence in a one-way ANOVA, *F*(3, 101) = 0.93, *p* = .430. Similarly, in Experiment 3, the standardised SAARC slopes did not differ significantly between [O J R A] (β_1_ = −0.18, *n* = 26), [R A O J] (β_1_ = −0.40, *n* = 25), [L E W U] (β_1_ = −0.49, *n* = 29), and [U L E W] (β_1_ = −0.05, *n* = 27), when testing the main effect of the between-subject factor letter sequence in a one-way ANOVA, *F*(3, 103) = 2.05, *p* = .11.

### Comparison of the OPE between letter sets

**Figure 6.**
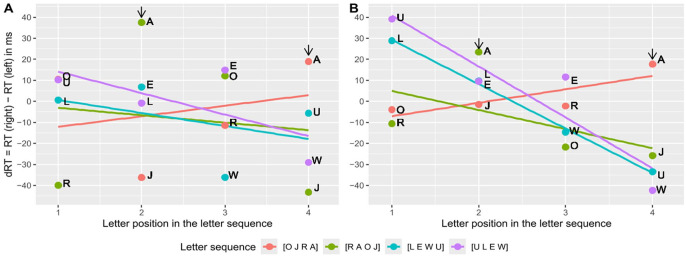
Differences in reaction times (dRT) with the right and left hand depending on the position of the letter in the memorised sequence (OPE) and averaged over participants in Experiment 2 (maintenance condition, Panel A) and Experiment 3 (retrieval condition, Panel B), for each letter set separately. The outlier letter A is marked with an arrow.

To examine the OPE in more detail, we also calculated the regressions per learned letter sequence separately. Figure 6 illustrates the resulting average dRTs per position in the letter sequence and the OPE slopes for Experiments 2 and 3. As reflected by the OPE being significant only in Experiment 3 and not in Experiment 2, the slopes are descriptively more negative in Experiment 3 than in Experiment 2 (cf. Figure 5, Panels B and A, respectively). Interestingly, in contrast to the SAARC slopes, not all OPE slopes are descriptively negative. Moreover, visual inspection suggests that the OPE differs depending on the used letter set (i.e., more negative OPE slopes for E, L, U, W than for A, J, O, R), and more precisely on the learned letter sequence (i.e., positive OPE slopes for [O J R A]). Hence, we conducted a series of additional one-sided *t-*tests with α = .05 to test the standardised OPE slopes against zero separately per letter sequence. In Experiment 3, only the OPE for [U L E W] and [L E W U] turned out significant, *t*(26) = 5.23, *p* < .001 and *t*(28) = 3.45, *p* = .001, respectively. None of the tests revealed a significant result in Experiment 2, all *p* ⩾ .061, although the relation between the slopes looks the same. In Experiment 2, the standardised OPE slopes differed significantly between the four memorised letter sequences [O J R A] (β_1_ = 0.32, *n* = 22), [R A O J] (β_1_ = −0.10, *n* = 22), [L E W U] (β_1_ = −0.18, *n* = 32), and [U L E W] (β_1_ = −0.27, *n* = 29), when testing the main effect of the between-subject factor letter sequence in a one-way ANOVA, *F*(3, 101) = 2.72, *p* = .048. Likewise, in Experiment 3, the standardised OPE slopes differed significantly between [O J R A] (β_1_ = 0.23, *n* = 26), [R A O J] (β_1_ = −0.09, *n* = 25), [L E W U] (β_1_ = −0.60, *n* = 29), and [U L E W] (β_1_ = −0.54, *n* = 27), *F*(3, 103) = 8.23, *p* < .001. Descriptively, the most negative unstandardized slopes were revealed with [U L E W], next [L E W U], then [R A O J], and positive slopes were only revealed with [O J R A] in both Experiments 2 and 3, which is visible in Figure 6. The absence or at least weaker presence of the OPE for [R A O J] and [O J R A] is noticeable. A closer look at Figure 6 can provide an explanation for this: dRTs for the letter A are higher than predicted by the position in the letter sequence for both letter sequences in both experiments and therefore have a high leverage. High dRTs for the letter A indicate that it is judged much faster with the left hand, regardless of its position within the letter sequence, which affects the estimation of the OPE. Note that we decided not to recalculate the OPE and SAARC slopes while excluding the dRT for letter A, because a regression slope should not be based on as few as three datapoints and because letter A only appeared in positions 2 and 4 but not 1 and 3 within the memorised sequences.

### Comparison between experiments

In an additional exploratory analysis, we compared the standardised SAARC effect between Experiments 1, 2, and 3, and the standardised OPE between Experiments 2 and 3 (uncorrected α = .05). First, a one-sided Jonckheere–Terpstra test for monotone trends was run to find out whether the SAARC effect based on LTM was strongest with no WM salience (Experiment 1), intermediate with intermediate WM salience (Experiment 2), and weakest with highest WM salience (Experiment 3). No evidence was found for this hypothesis, *JT* = 22667, *p* = .122. Second, a one-sided independent-samples Welch *t*-test was computed to investigate whether the OPE was stronger with high WM salience (Experiment 3) than with intermediate WM salience (Experiment 2). Our data supported this hypothesis, *t*(209.65) = 1.67, *p* = .049. In addition, in one ANOVA per letter sequence, we explored whether WM salience affected spatial response preferences for different letters to different extents, but the interaction for dRTs between experiments and letters was not significant in any of the four-letter sequences (see the online Supplementary Material D).

### Correlation between the SAARC effect and the OPE

Moreover, we investigated whether the SAARC effect and the OPE correlate with one another. Our data revealed no evidence of a correlation between the effects. For Experiment 2, the correlation remained non-significant, *r* = 0.02, *t*(103) = 0.21, *p* = .832, and so did the correlation for Experiment 3, *r* = 0.07, *t*(105) = 0.77, *p* = .443.

## General discussion

With this series of experiments, we investigated whether the account postulating a stable spatial long-term representation and the WM account postulating spatial short-term representations (van Dijck et al., 2014) can be transferred from numbers to other ordered stimuli such as letters. Previous literature has shown evidence for both accounts, and the SNARC effect and the OPE for numbers have even been observed simultaneously. Assuming similar mechanisms for mental spatial representations of letters as for those of numbers, a SAARC reflecting the alphabetical order stored in LTM was expected when no new ordinal sequence had to be learned or when a new ordinal sequence merely had to be maintained without being task-relevant. In contrast, an OPE for a new ordinal sequence stored in WM was hypothesised only when the sequence needed to be retrieved from WM to perform the task correctly.

The starting point was a control condition (Experiment 1), with which we wanted to test the suitability of our go/no-go design for the investigation of spatial response preferences for letters. Importantly, as compared to numbers which comprise information on both magnitude and order, letters have an ordered nature but no inherent magnitude characteristic. In line with previous results of Gevers et al. (2003), we found that participants responded faster to letters at the beginning of the alphabet with the left hand than with the right hand, while letters at the end of the alphabet were responded to faster with the right than with the left hand (i.e., a SAARC effect), despite the presence of “no-go” trials. This manipulation check provided a solid basis for the investigation of a maintenance condition (i.e., intermediate WM salience, Experiment 2) vs. a retrieval condition (i.e., high WM salience, Experiment 3) of previously memorised sequences during consonant-vowel classification on spatial associations. Here, on top of the inherent ordinality letters stemming from the alphabet stored in LTM, an artificial different ordinality was created in WM by the instruction to memorise a letter sequence. In line with the results of Ginsburg et al. (2014), in Experiment 2, we found no spatial response preference for the position of the letters in the memorised sequence (i.e., no evidence for an OPE). However, as in Experiment 1, we found a response preference for the position of the letters within the alphabet (i.e., a SAARC effect). In this case, the findings for numbers seem to be transferable to letters. Furthermore, also consistent with results by Ginsburg et al. (2014), we found an OPE in Experiment 3, in which the WM salience was high. Moreover, our exploratory analysis confirmed that the OPE was significantly stronger with high than with intermediate WM salience. This supports the conclusion of Ginsburg et al. (2014) and van Dijck et al. (2014) that retrieval during the task is crucial for the presence of an OPE and extends it to classification tasks of other ordinal stimuli than numbers, namely letters. Interestingly, however, one needs to consider that the ordinal position stored in LTM and thus the spatial mapping of some letters might be very robust and not be overruled by newly learned ordinal positions stored in WM.

These results have important implications for theories and explanations of spatial-numerical associations on the one hand and of spatial associations with non-numerical ordinal stimuli on the other. First, they provide evidence that ordinality (short- and long-term) can elicit spatial mapping in case no cardinality information is present. When participants need to memorise a sequence of arbitrary objects in WM (e.g., fruit and vegetable names; van Dijck & Fias, 2011) and recall the sequence during task execution, the WM ordering is decisive (i.e., there is no magnitude nor long-term memory order). In the case of overlearned ordinal sequences such as letters, the ordinality is crucial (i.e., there is no quantity or magnitude). Whether a sequence kept in LTM or a sequence kept in WM is decisive for the spatial mapping (i.e., when both long- and short-term ordinality are present) remained unclear. In the present study, we found that the salience of the WM order is decisive.

The SAARC effect reflecting spatial mappings of ordinality coming from LTM seems to be small. Specifically, the SAARC effect found by Gevers et al. (2003) had an unstandardized slope of 1.12 ms. The unstandardized slopes in the current study were similar (i.e., ‒1.65 ms in Experiment 1, ‒1.93 ms in Experiment 2, and ‒1.78 ms in Experiment 3). This might even have led Dehaene and colleagues (1993) to rule out the role of ordinality in LTM for creating spatial-numerical associations (see their Experiment 4). Due to its rather small effect sizes, a true SAARC effect might have simply gone undetected with only 10 participants. However, the spatial response preferences we found for letters in Experiment 1 support the hypothesis that spatial associations could be the result of overlearned sequences and thus not be unique to numerical stimuli. Our results are in line with the results by Gevers et al. (2003) showing spatial response preferences for non-numerical ordinal stimuli, namely for letters or months.

At first sight, the present results support both the LTM and the WM account (van Dijck & Fias, 2011; see also Abrahamse et al., 2014, 2016; van Dijck et al., 2014). The WM account assumes that spatial associations are the result of the ordinal position of the stimuli in the WM, with stimuli at the beginning of the memorised sequence being associated with the left and stimuli at the end of the sequence being associated with the right. In the present study, an OPE was detected (i.e., a spatial response preference depending on the ordinal position of the stimuli in WM), investigated via letter sequences memorised before the consonant-vowel classification. This was analogous to van Dijck and Fias’ original study design, with number sequences memorised before parity judgement, but opposed to Koch et al.’s (2023) design, where inherent ordinal information of numbers was used, and no number sequences had to be memorised before the task. Since not only numerical stimuli can be memorised in an artificial order in WM, the WM approach can explain not only the SNARC effect but also spatial associations of non-numerical ordinal stimuli, like in our case letters. However, we only found the OPE if the memorised letters had to be retrieved during the consonant-vowel classification task (retrieval condition) but not if they were only held in memory for later recall (maintenance condition), although we found a SAARC effect reflecting long-term associations. This pattern of results is consistent with the results of Ginsburg et al. (2014) who identified retrieval during the classification task as a prerequisite for the occurrence of the OPE and concluded that the spatial associations between the ordinal stimuli in WM and response arise during the classification itself when the information is retrieved. Importantly, these findings can be interpreted in two ways: First, when no retrieval of the WM content is required by the task, this could either result in the ordinality information of the WM content not being activated, while the stored items are activated. Second, it could result in the WM content not being activated at all and being only passively stored.

Interestingly, we also found a coexistence of the OPE and the SAARC effect in the retrieval condition (Experiment 3). These results are in line with studies by Ginsburg and Gevers (2015) and Huber et al. (2016), who have already shown the coexistence of the OPE and the SNARC effect for numerical stimuli. The two simultaneously arising effects have been interpreted as the result of different representations, with the SNARC effect being based on the representation of numbers and their respective canonical order in LTM, and the OPE being based on the spatial short-term bindings in WM, which are formed during retrieval (Ginsburg and Gevers, 2015; Huber et al., 2016; van Dijck et al., 2015). This hybrid explanatory approach for numbers seems to be applicable to non-numerical ordered stimuli such as letters and can therefore also account for our results: The SAARC effect, which is the result of the alphabetical order stored in LTM, can be overcome by an OPE, which reflects short-term spatial associations based on sequences stored in WM if certain conditions are fulfilled (e.g., necessity of retrieval of the learned sequence during the task). Note that the coexistence of the two effects within one experiment could be driven by different trials instead of them influencing response patterns simultaneously. As recently demonstrated by Ftaïta et al. (2024), spatialization of ordinal information coming from a newly learned sequence stored in WM vanishes over time when the sequence is repeated. A potential explanation offered by the authors is that spatialization gets weaker and finally disappears with the newly learned sequence being chunked and moved from WM to LTM. In our design, where the encoding phase is followed by a relatively long classification phase, the OPE could be dominant in the first trials, where the newly learned sequence is still very present in WM, and the SAARC could be dominant in the later trials, where the sequence fades into the background. This would be in line with Ftaïta et al.’s findings; however, it is not possible to investigate this in the present study. In both their and our study, the response-to-key assignment was switched after the first half, and the OPE was calculated as a regression of the differences in RTs between left and right responses on the ordinal position. Crucially, Ftaïta et al. introduced a new ordinal sequence before the second half of their experiment, which is why both left and right responses might equally be affected by spatialization based on ordinal positions. In contrast, in the present study, only one sequence had to be remembered for both response-to-key assignments together, which is why the spatialization might only have occurred in the first trials with one response key but not in the first trials with the respective other. Although for these reasons changes in the OPE over the course of the experiment cannot be tested within the present study, another argument for the OPE vanishing over time in Ftaïta et al.’s study is that it was only task-relevant *which* elements were part of the memorised sequence, whereas their order was not task-relevant. This is an important difference to van Opstal et al.’s (2009) experiment and to two of Previtali et al.’s (2010) experiments, where participants were to make order judgements. Hence, the ordinal information might get lost over time, which is not the case for the canonical order stored in LTM.

### Stimulus-specific effects on short-term and long-term letter-space associations

Regarding numbers, it has been known for a long time that zero has a special role for spatial-numerical associations (Nuerk et al., 2004; Pinhas & Tzelgov, 2012). Its mental representation differs from those of other numbers (Nieder, 2016), leading to observations such as its parity status being unclear to many students (e.g., Levenson et al., 2007). Uncertainty in parity judgement for number zero can therefore impair the estimation of the SNARC effect (Huber et al., 2016; Nuerk et al., 2004; Roth et al., 2025b). Our results suggest that such stimulus-specific effects can be found for letters as well and that they need to be considered in interpretations. Some letters seem to have a very strong long-term spatial association so that short-term associations do not play a role for them. At the same time, for many other letters, associations in both LTM and WM seem to influence responses. As the WM account did not propose different statuses of different elements within a specific sequence, our design was not prepared to test such effects in a systematic fashion. However, our observations call for such investigations in future studies.

From what we could see in our study, responses were descriptively faster for the letter A than for any other letter (see Table B1 in the online Supplementary Material B). Moreover, the letter A was answered faster with the left hand than with the right hand regardless of its position in the memorised sequence. We can speculate that this pattern could be explained by an especially strong, immediately available long-term representation of the letter A as the first letter of the alphabet in LTM. This leading to the respective spatial association and response preference with the left side might have exceeded the interfering activation of the spatial association and response preference based on the short-term representation of the memorised letter sequence in WM. Faster responses with the left for the letter A, regardless of its position in the memorised letter sequence, might be the reason for the OPE to differ between letter sequences, as can be seen in Figure 6 in the reversed slope for [O J R A]. Note that Gevers et al. (2003) did not use letter A in their study. Our findings could indicate that the OPE can be overridden by automated, highly overlearned stimulus associations. As outlined above, the influence of individual stimuli on spatial response behaviour has also been observed for numbers before. These data are the first to show such stimulus-specific influences for spatial LTM and WM associations for letters. Future research should investigate to what extent overlearned associations from items stored in the LTM can override short-term associations relying on sequences stored in WM on the one hand, and to what extent short-term associations can override long-term associations on the other hand. For instance, are influences of spatial long-term associations and seemingly facilitated processing limited to A as the first letter of the alphabet, or is there an analogous effect for the letter Z as the last letter of the alphabet? Here, one would expect a strong spatial association with the right. It would also be interesting to see whether a letter-specific effect is also evident for neighbouring letters such as B and C or X and Y. Moreover, training studies could investigate how newly created but strongly trained associations affect the OPE.

The fact that some letters seem to have rather stable LTM representations, whereas LTM representations can be more easily overruled by WM content for other letters, can also explain that no correlation was found between the OPE and the SAARC effect. However, apart from stimulus-specific effects, there are at least two more reasons for the lack of evidence for a correlation: The experimental task might have low test–retest reliability, or the OPE and the SAARC effect might just vary and be unstable on an intraindividual level. This seems to be the case for the SNARC effect (Roth et al., 2024) and be similar for the OPE and SAARC effect.

### Modal and amodal representations of numbers, letters, and their spatialization

Kaup et al. (2024) suggest a major distinction of mental representations between modal (i.e., modality-specific) and amodal (i.e., modality-unspecific) representations. They also suggest that explanatory approaches for the SNARC effect can be categorised into modal and amodal approaches. According to the view proposed by Dehaene et al. (1993), the SNARC effect is seen as the result of modal magnitude representations specific to numbers, which are arranged on the MNL aligned from left-to-right in Western cultures due to modal experiences of reading and writing direction. According to ATOM (Bueti & Walsh, 2009; Walsh, 2003), this modal account magnitude dimensions other than numbers (e.g., physical size or luminance; Cohen Kadosh et al., 2008; Fumarola et al., 2014; Ren et al., 2011). According to Pitt and Casasanto (2018), this holds only for the modality of prothetic dimensions (i.e., where humans experience quantitative variations) that entail quantity or magnitude, but not for metathetic dimensions (i.e., where humans experience qualitative variations). Conversely, the WM account by van Dijck and Fias (2011) can be seen as an amodal (i.e., unspecific and not only applicable to quantity or magnitude dimensions, but also other items) approach, as it can explain spatial response preferences for very different kinds of stimuli, including objects without any inherent magnitude or ordinality structure, such as fruits and vegetables.

The current data, especially from Experiment 3, confirm the notion that both modal and amodal representations can contribute to letter-space associations, similar to spatial-numerical associations (see also Koch et al., 2023, for numbers). Notably, the current data suggest that the influence of modal and amodal representations on the spatial mental mapping of letters may also depend on individual letter identity and learning. For example, a strong association of A with the left could occur because this letter being the first in the alphabet is highly overlearned, both visually and auditorily. For instance, in German, learning the alphabet at school is colloquially usually referred to as “learning the ABC,” so A comes first even in the colloquial name of the alphabet. This highly overlearned standard visual and auditory input and output may explain the strong long-term associations and the weak or even non-existent short-term association for the letter A. Likewise, a recent study has shown that the spatial association of number 1 with the left is stronger than what would be expected when deriving predictions from the regression line fit to all numbers within the stimulus set where number 1 is the smallest number, or when comparing with the spatial association of the lowest number in other stimulus sets (Roth et al., 2025b). Future studies should pit spatial associations resulting from WM or LTM against each other by administering sequences to be memorised by participants in which the stimuli have the opposite artificial order as compared to their canonical order.

### Limitations of the present study

Despite our recruitment efforts, the sample size required according to the preregistered power analysis was not achieved. However, 169 participants took part in the maintenance condition (Experiment 2) and another 168 in the retrieval condition (Experiment 3), which largely exceeds the sample sizes used within the published literature testing the OPE (e.g., Ginsburg et al., 2014; Ginsburg & Gevers, 2015; van Dijck & Fias, 2011) and the SAARC effect (Gevers et al., 2003). This still makes our samples the largest that has ever been used to investigate the OPE with letters and the SAARC effect (note, however, that previous studies have administered several ordinal sequences to be memorised, whereas there was only one such sequence in our experiments). These sample sizes permitted us to observe a significant OPE at least in Experiment 3 and a significant SAARC effect in all three Experiments (see Table 4).

The present study was conducted online, which means that—in contrast to a laboratory study—it was not possible to keep all conditions constant for all participants. To be able to exclude data from participation with distractions (such as noise), quality items were assessed at the end of the study. Based on these quality items, we excluded datasets from participants who indicated that they performed the experiment in a noisy environment or that they would not use their data if they were the experimenters. Importantly, the SNARC effect can be observed reliably in online settings and is comparable to laboratory settings (e.g., Cipora et al., 2019; Gökaydin et al., 2018; Koch et al., 2023; Roth et al., 2025b; Roth et al., 2025a), so we assume that this is the case for the OPE and the SAARC effect, too.

We also asked the participants how they memorised the letter sequence and whether they had noted it down, implying that they had not necessarily stored the letter sequence in their WM but rather externalised it. In case participants noted down the letter sequence, neither maintenance of the memorised letter sequence nor retrieval from WM during the classification task can be ensured, which is why respective participants were excluded. In addition to these potential difficulties, the online setting does offer various advantages, such as the collection of a larger and more diverse sample. This increases representativeness and generalisability of the results as compared to standard laboratory experiments with university students (Reips, 2002). Nevertheless, it should be mentioned that the present results are limited to cultures with a left-to-right reading and writing direction, which cannot easily be transferred to cultures with a different reading and writing directions (Bulut et al., 2025; Hochman et al., 2025; Shaki et al., 2009; Shaki & Gevers, 2011; Zebian, 2005).

Importantly and as opposed to many previous studies, there was only one encoding phase in Experiments 2 and 3 of the present study, such that participants completed 320 trials with the same letters determining the go/no-go rule. One could argue that after a certain number of trials in Experiment 3, participants might have generated an episodic memory trace of the task execution and know which letters to respond to and which not. That is, they might not need to retrieve the ordered sequence stored in WM anymore after a certain number of trials so that the order of the sequence does not affect the response patterns anymore in later trials. Moreover, the position of each letter in LTM and in WM was not fully orthogonal in the present study, because all letters have a fixed position in LTM but were only presented on two instead of four potential positions within the ordinal sequence stored in WM (e.g., the letter O was on position 1 in participants administered with [O J R A] and on position 3 for participants administered with [R A O J], but never on positions 2 and 4). Therefore, the current design is not ideal for the investigation of the interactions between letter positions in LTM and WM. However, 20 repetitions per combination of stimulus and response-to-key assignment permitted us a very precise estimate of the SAARC effect and of the OPE, which was a clear advantage of the current study’s design with a relatively long classification phase.

## Conclusion

The current study demonstrates the coexistence of long-term letter-space associations (i.e., stimulus-specific for the letter A and the SAARC effect) and short-term stimulus-unspecific associations (i.e., the OPE produced by a letter sequence stored in WM). These two representations do not seem to be mutually exclusive (see also Ginsburg & Gevers, 2015). While the first point towards modal spatial mappings for letters (i.e., letters early in the alphabet are associated with the left and letters late in the alphabet are associated with the right), the latter point towards amodal stimulus-unspecific spatial mappings (i.e., stimuli early in a memorised sequence are associated with the left and stimuli late in a memorised sequence are associated with the right). Importantly, not only did we thereby replicate the findings by Gevers et al. (2003), but we were also able to extend the findings by Ginsburg et al. (2014) from numbers to letters as a different type of ordinal stimuli. We found that mere maintenance of a memorised sequence is not sufficient to observe an OPE; instead, retrieval during the classification task is crucial. Our findings point towards the possibility that overlearned orders and their spatial associations in LTM can coexist with newly learned orders and their spatial short-term associations in WM, thus providing evidence for a hybrid model that integrates both modal and amodal representations.

## Supplemental Material

sj-docx-1-qjp-10.1177_17470218251324437 – Supplemental material for Looks like SNARC spirit: Coexistence of short- and long-term associations between letters and space Supplemental material, sj-docx-1-qjp-10.1177_17470218251324437 for Looks like SNARC spirit: Coexistence of short- and long-term associations between letters and space by Lilly Roth, Julia F. Huber, Sophia Kronenthaler, Jean-Philippe van Dijck, Krzysztof Cipora, Martin V. Butz and Hans-Christoph Nuerk in Quarterly Journal of Experimental Psychology

## Notes

1. A recent study by Vivion et al. (2024) showed that sequences of four items are most suitable to detect spatialization.

2. Bonferroni correction was preregistered, but we decided to apply Bonferroni-Holm correction, because it represents a better balance between type I and type II errors and is a generally more recommended method. At the same time, in the present study, the significance of all results would remain unchanged if the Bonferroni method was used.

3. When excluding this participant from the main analyses, the results (see below) did not change: The SAARC effect remained significant, β_1_ = 0.38, *t*(103) = 5.95, *p* < .001, and the OPE remained non-significant, β_3_ = 0.09, *t*(103) = 1.11, *p* = .136. Hence, we did not exclude this participant’s data from any data analyses reported in this manuscript.

4. When excluding this participant from the main analyses, the results (see below) did not change: The SAARC effect, β_1_ = 0.28, *t*(105) = 3.87, *p* < .001, and the OPE remained significant, β_3_ = 0.27, *t*(105) = 3.55, *p* < .001. Hence, we did not exclude this participant’s data from any data analyses reported in this manuscript.
