# Supplementary material for: Looks like SNARC spirit: Coexistence of short- and long-term associations between letters and space
Source: Q J Exp Psychol (Hove). 2025 Feb 17;78(10):2110–32. doi: 10.1177/17470218251324437 (PMC12432287; doi:10.1177/17470218251324437)
Supplement: sj-docx-1-qjp-10.1177_17470218251324437 – Supplemental material for Looks like SNARC spirit: Coexistence of short- and long-term associations between letters and space [file sj-docx-1-qjp-10.1177_17470218251324437.docx]

Supplementary Material for:

**Looks like SNARC spirit:**

**Coexistence of short- and long-term associations between letters and space**

Lilly Roth^1^*, Julia F. Huber^1^*, Sophia Kronenthaler^1^, Jean-Philippe van Dijck^2,3^, Krzysztof Cipora^4^, Martin V. Butz^5^, Hans‑Christoph Nuerk^1,6,7^

^1^ Department of Psychology, University of Tübingen, Tübingen, Germany

^2^ Department of Applied Psychology, Thomas More University, Antwerp, Belgium

^3^ Department of Experimental Psychology, Ghent University, Ghent, Belgium

^4^ Centre for Mathematical Cognition, Loughborough University, Loughborough, UK

^5^ Department of Computer Science, University of Tübingen, Tübingen, Germany

^6^ LEAD Graduate School & Research Network, University of Tübingen, Tübingen, Germany

^7^ German Center for Mental Health (DZPG), Berlin, Germany

* equal contribution, shared first-authorship

**E-mail-addresses:** [lilly.roth@uni-tuebingen.de](mailto:lilly.roth@uni-tuebingen.de), [julia.huber@psycho.uni-tuebingen.de](mailto:julia.huber@psycho.uni-tuebingen.de), [sophia.kronenthaler@gmail.com](mailto:sophia.kronenthaler@gmail.com), [jean-philippe.vandijck@thomasmore.be](mailto:jean-philippe.vandijck@thomasmore.be), [k.cipora@lboro.ac.uk](mailto:k.cipora@lboro.ac.uk), [martin.butz@uni-tuebingen.de](mailto:martin.butz@uni-tuebingen.de), [hc.nuerk@uni-tuebingen.de](mailto:hc.nuerk@uni-tuebingen.de)

**Corresponding author:** Prof. Dr. Hans-Christoph Nuerk

**Supplementary Material A: Stimulus selection**

When selecting the letters to be used as stimuli in Experiments 1, 2, and 3, and when selecting the letter sequences to be learned in Experiments 2 and 3, we took various factors into account. Specifically, (1) the length of the sequences, (2) the letters within the sequences, (3) the order of the letters, and (4) the choice of consonants versus vowels were considered as described in detail below:

**(1) Length of the letter sequences**

The working memory seems to have a capacity limit between three and five stimuli (Cowan, 2001, 2010). To ensure that participants could successfully repeat the letter sequences memorize the letter sequences, we chose a length of the letter sequences of four letters. Additionally, a recent study by Vivion et al. (2024) further validated that a sequence length of four items is suitable to detect spatialization.

**(2) Selection of the letters within the sequences**

The experiment should be carried out using a computer keyboard. On a computer keyboard, certain keys (letters) are pressed with the right or left hand, depending on the writing style (Feit et al., 2016). It is possible that experience with certain keys generates spatial response behavior. For an overview of the locations of the letters used in the present study on typical German and English computer keyboards, see Figure 1 in the main manuscript. For example, the letter O might be judged more quickly with the right hand, as the key with the letter O is regularly pressed with the right hand. In order to keep this potential effect as constant as possible, only letters were selected that are pressed exclusively with the right or left hand, regardless of writing style (Feit et al., 2016). Moreover, the letters Y and Z were excluded because they have different positions on a German and English keyboard. The letters D and K were excluded as these were to be used as response keys. From the 11 remaining consonants (C, F, J, L, M, P, Q, R, S, W, X) and five vowels (A, E, I, O, U), this results in 13200 possible letter sequences consisting of two different consonants and vowels.

**(3) Selection of the order of the letters within the sequences**

The order of the letters in these 13200 possible sequences can be ascending (e.g., A C E F), descending (e.g., F E C A), and random (C A F E). According to Lindemann et al. (2008), memorizing a descending number sequence can reduce the size of the SNARC effect. Ascending letter sequences make it more difficult to distinguish between a SNARC-like effect for letters and the ordinal position effect. Therefore, we chose random sequences.

**(4) Considerations regarding consonants and vowels**

We made sure that each letter sequence contained a consonant and a vowel that are regularly typed with the right and left hand respectively (Feit et al., 2016). There is also the possibility that participants react more quickly to consonants or vowels with the right or left hand, regardless of the letters (MARC effect; see e.g., Cipora et al., 2019). To balance out this possible effect, no letter sequences were selected that begin or end with two vowels or consonants (like [E U L W] or [R J A O]), leading to 2596 remaining possible letter sequences.

Next, the area of the alphabet covered by the letter sequences should be as large as possible (total spread), and the letter sequence should be as evenly spaced as possible (distance measure). For instance, for [P X I A] all letters were sorted according to their alphabetical order (i.e., A, I, P, X). Then, each letter was assigned a number based on the position of the letters in the alphabet (i.e., A = 1, I = 9, P = 16, X = 24). Next, the distances between the consecutive letters were calculated by the absolute value of the position difference (i.e., |1-9| = 8, |9-16| = 7, |16 - 24| = 8). From the resulting triple (i.e., 8, 7, 8), the sum of the distances (i.e., 8 + 7 + 8 = 21) can be calculated to estimate the total spread, and the sum of the differences of the distances to each other (i.e., |8-7| + |8-8| + |7-8| = 2) provides a distance measure.

We excluded all letter sequences that have neighboring letters or a distance of one. For the remaining 2156 letter sequences the correlation between the total spread and the distance measure is *r* = 0.6. Letter sequences with a high total spread therefore also have a high distance measure and vice versa. This means that no letter sequence with maximum spread and minimum distance measure could be selected. Instead, letter sequences as close as possible to the center of the joint distribution were selected by choosing letter sequences with a Mahalanobis distance less than or equal to one. The selection procedure based on the Mahalanobis distance was carried out twice; in the third run, no further letter sequences with a distance less than or equal to one could be selected. The first selection step led to a reduction to 814 letter sequences, the second to a reduction to 154 letter sequences. The remaining 154 letter sequences were divided into a total of seven letter sets each in 22 different orders. The final two sets of letters were subjectively selected with two orders: [O J R A], [R A O J], [L E W U], and [U L E W]. Care was taken to ensure that the letters did not form a known word or an abbreviation in order to avoid memorization of the letter sequences by means of chunking (Gobet et al., 2001).

In Experiment 1 and 2, the following symbols were selected as ‘no-go’ stimuli: ‡, △, ≡, and ∇. When making the selection, we ensured not to use symbols with already assigned semantics (e.g., &, %). In addition, the symbols should be as similar as possible to the letters used.

**References for Supplementary Material A**

Cipora, K., Soltanlou, M., Reips, U.‑D. & Nuerk, H.‑C. (2019). The SNARC and MARC effects measured online: Large-scale assessment methods in flexible cognitive effects. *Behavior Research Methods*, *51*(4), 1676–1692. <https://doi.org/10.3758/s13428-019-01213-5>

Cowan, N. (2001). Metatheory of storage capacity limits. *Behavioral and Brain Sciences*, *24*(1), 154–176. <https://doi.org/10.1017/s0140525x0161392x>

Cowan, N. (2010). The Magical Mystery Four: How is Working Memory Capacity Limited, and Why? *Current Directions in Psychological Science*, *19*(1), 51–57. <https://doi.org/10.1177/0963721409359277>

Feit, A. M., Weir, D. & Oulasvirta, A. (2016). How We Type. In J. Kaye, A. Druin, C. Lampe, D. Morris & J. P. Hourcade (Eds.), *Proceedings of the 2016 CHI Conference on Human Factors in Computing Systems* (pp. 4262–4273). ACM. <https://doi.org/10.1145/2858036.2858233>

Gobet, F., Lane, P. C. R., Croker, S., Cheng, P. C.‑H., Jones, G., Oliver, I. & Pine, J. M. (2001). Chunking mechanisms in human learning. *Trends in Cognitive Sciences*, *5*(6), 236–243. <https://doi.org/10.1016/S1364-6613(00)01662-4>

Lindemann, O., Abolafia, J. M., Pratt, J. & Bekkering, H. (2008). Coding strategies in number space: memory requirements influence spatial-numerical associations. *Quarterly Journal of Experimental Psychology, 61*(4), 515–524. <https://doi.org/10.1080/17470210701728677>

**Supplementary Material B**

**Table B2**

*Mean accuracies of responses for ‘go’ trials per letter set / sequence and per experiment*

| Letter set */ sequence* | Experiment 1 | Experiment 2 | Experiment 3 |
| --- | --- | --- | --- |
| [O J R A] | 0.924 | 0.957 | 0.923 |
| [R A O J] |  | 0.960 | 0.948 |
| [L E W U] | 0.942 | 0.959 | 0.937 |
| [U L E W] |  | 0.938 | 0.945 |

**Supplementary Material C**

**Table C1**

*Literature overview for studies that have investigated the role of magnitude vs. ordinality in spatial mental representations, with WM demands referring to the WM task, and with magnitude relevance (only for number tasks), ordinality relevance (either of sequences stored in LTM or in WM), and WM salience (referring to whether a sequence stored in WM is made salient within the judgment task), ordered chronologically by publication date.*

| Study and experiment | Stimuli and judgment task | WM demands | Nature of task | Magnitude relevance  (stored in LTM) | Ordinality relevance  (stored in LTM or WM) | WM salience | Observed effect(s) |
| --- | --- | --- | --- | --- | --- | --- | --- |
| Gevers et al. (2003), Exp. 1 | Months: before vs. after July | None | Respond to all trials | No magnitude | LTM order relevant  (direct task),  no WM order | None | SNARC-like effect (LTM) |
| Gevers et al. (2003), Exp. 1 | Months: ends with the letter R vs. not | None | Respond to all trials | No magnitude | LTM order irrelevant  (indirect task),  no WM order | None | SNARC-like effect (LTM) |
| Gevers et al. (2003), Exp. 2 | Letters: before vs. after the letter O | None | Respond to all trials | No magnitude | LTM order relevant  (direct task),  no WM order | None | SNARC-like effect (LTM) |
| Gevers et al. (2003), Exp. 2 | Letters: consonant vs. vowel | None | Respond to all trials | No magnitude | LTM order irrelevant  (indirect task),  no WM order | None | SNARC-like effect (LTM) |
| Gevers et al. (2004) | Days of the week: before vs. after Wednesday | None | Respond to all trials | No magnitude | LTM order relevant  (direct task),  no WM order | None | SNARC-like effect (LTM) |
| Gevers et al. (2004) | Days of the week: contains letter R vs. not | None | Respond to all trials | No magnitude | LTM order irrelevant  (indirect task),  no WM order | None | SNARC-like effect (LTM) |
| Van Dijck & Fias (2011), Exp. 1 | Number parity: odd vs. even | Sequence stored in WM | Go/no-go design: only respond to numbers from sequence stored in WM | Magnitude irrelevant (indirect task) | LTM order irrelevant  (indirect task),  WM order irrelevant  (indirect task) | High | OPE (WM), but no evidence for SNARC effect (LTM) |
| Van Dijck & Fias (2011), Exp. 2 | Word meaning: fruit vs. vegetable | Sequence stored in WM | Go/no-go design: only respond to numbers from sequence stored in WM | No magnitude | LTM order irrelevant  (indirect task),  WM order irrelevant  (indirect task) | High | OPE (WM) |
| Ginsburg et al. (2014), Exp. 1 | Number parity: odd vs. even | Sequence stored in WM | Go/no-go design: only respond to numbers from sequence stored in WM | Magnitude irrelevant (indirect task) | LTM order irrelevant  (indirect task),  WM order irrelevant  (indirect task) | High | OPE (WM), but no evidence for SNARC effect (LTM) |
| Ginsburg et al. (2014), Exp. 2 | Number magnitude: small vs. large | Sequence stored in WM | Go/no-go design: only respond to numbers from sequence stored in WM | Magnitude relevant (direct task) | LTM order relevant  (direct task), WM order irrelevant  (indirect task) | High | OPE (WM), but no evidence for SNARC effect (LTM) |
| Ginsburg et al. (2014), Exp. 3 | Number parity: odd vs. even | Sequence stored in WM | Respond to all trials | Magnitude irrelevant (indirect task) | LTM order irrelevant  (indirect task),  WM order irrelevant  (indirect task) | Intermediate | SNARC effect (LTM), but no evidence for OPE (WM) |
| Ginsburg et al. (2014), Exp. 4 | Number magnitude: small vs. large | Sequence stored in WM | Respond to all trials | Magnitude relevant (direct task) | LTM order relevant (direct task),  WM order irrelevant  (indirect task) | Intermediate | SNARC effect (LTM), but no evidence for OPE (WM) |
| Guida et al. (2015) | Letter probe recognition: part of the auditory sequence vs. not | Sequence stored in WM | Respond to all trials | No magnitude  LTM order irrelevant | (indirect task),  WM order irrelevant  (indirect task) | High | OPE (WM), but SNARC-like effect (LTM) not tested |
| Huber et al. (2016) | Number parity: odd vs. even | Sequence stored in WM | Go/no-go design: only respond to numbers from sequence stored in WM | Magnitude irrelevant (indirect task) | LTM order irrelevant  (direct task),  WM order irrelevant  (indirect task) | High | Both SNARC effect (LTM) and OPE (WM) |
| Current study, Exp. 1 | Letters: consonant vs. vowel | None | Go/no-go design: only respond to numbers | No magnitude | LTM order irrelevant (indirect task),  WM order irrelevant  (indirect task) | None | SAARC effect (LTM), but no evidence for OPE (WM) |
| Current study, Exp. 2 | Letters: consonant vs. vowel | Sequence stored in WM | Go/no-go design: only respond to numbers | No magnitude | LTM order irrelevant (indirect task),  WM order irrelevant  (indirect task) | Intermediate | SAARC effect (LTM), but no evidence for OPE (WM) |
| Current study, Exp. 3 | Letters: consonant vs. vowel | Sequence stored in WM | Go/no-go design: only respond to numbers from sequence stored in WM | No magnitude | LTM order irrelevant (indirect task),  WM order irrelevant  (indirect task) | High | Both SAARC effect (LTM) and OPE (WM) |

**References for Supplementary Material C**

Gevers, W., Reynvoet, B., & Fias, W. (2003). The mental representation of ordinal sequences is spatially organized. *Cognition*, *87*(3), 87–95. <https://doi.org/10.1016/S0010-0277(02)00234-2>

Gevers, W., Reynvoet, B., & Fias, W. (2004). The mental representation of ordinal sequences is spatially organized: evidence from days of the week. *Cortex; a Journal Devoted to the Study of the Nervous System and Behavior*, *40*(1), 171–172. <https://doi.org/10.1016/S0010-9452(08)70938-9>

Ginsburg, V., van Dijck, J.-P., Previtali, P., Fias, W., & Gevers, W. (2014). The impact of verbal working memory on number-space associations. *Journal of Experimental Psychology: Learning Memory and Cognition*, *40*(4), 976–986. <https://doi.org/10.1037/a0036378>

Guida, A., Leroux, A., Lavielle-Guida, M., & Noël, Y. (2015). A SPoARC in the dark: Spatialization in verbal immediate memory. *Cognitive Science, 40*(8), 2018–2121. <https://doi.org/10.1111/cogs.12316>

Huber, S., Klein, E., Moeller, K., & Willmes, K. (2016). Spatial-Numerical and ordinal positional associations coexist in parallel. *Frontiers in Psychology*, *7*:438, 1–13. <https://doi.org/10.3389/fpsyg.2016.00438>

van Dijck, J.-P., & Fias, W. (2011). A working memory account for spatial–numerical associations. *Cognition*, *119*(1), 114–119. <https://doi.org/10.1016/j.cognition.2010.12.013>

**Supplementary Material D**

**Comparisons of dRTs between letters and experiments**

To explore whether WM salience (which was higher in Experiment 3 than in Experiment 2) affected spatial response preferences for some letters more than for others, we ran a two-way mixed ANOVA for dRT separately for each memorized sequence. Each of the four ANOVAs considered one between-subjects factor (WM salience: intermediate in Experiment 2 vs. high in Experiment 3) and one within-subjects factor (letter: A vs. J vs. O vs. R when the memorized sequence was [O J R A] or [R A O J], and E vs. L vs. U vs. W when the memorized sequence was [L E W U] or [U L E W]). A Bonferroni-Holm correction was applied for the interpretation of the four ANOVA outcomes, starting with a significance level of *α* = .05 / 4 = .0125 for the lowest *p*‑value.

Most importantly, the interaction effect between WM salience and memorized sequence remained non-significant for each letter sequence ([O J R A]: *F*(3, 138) = 1.27, *p* = .287; [R A O J]: *F*(3, 135) = 2.21, *p* = .089; [L E W U]: *F*(3, 177) = 1.91, *p* = .130; [U L E W]: *F*(3, 162) = 0.967, *p* = .410). In other words, we did not find evidence for the stability (in terms of robustness against increasing salience of ordinality stored in WM) of spatial response preferences to be stronger for some letters than for others. Moreover, no significant main effect of WM salience was found (averaged over letters), pointing towards similar dRTs in Experiments 2 and 3 ([O J R A]: *F*(1, 46) = 0.926, *p* = .341; [R A O J]: *F*(1, 45) = 0.001, *p* = .971; [L E W U]: *F*(1, 59) = 0.816, *p* = .370; [U L E W]: *F*(1, 54) = 0.541, *p* = .465). However, spatial response preferences differed between letters in each of the four letter sequences (averaged over both Experiments 2 and 3), as revealed by a significant main effect of letter in each of the four ANOVAs ([O J R A]: *F*(3, 138) = 2.68, *p* < .050; [R A O J]: *F*(3, 135) = 8.48, *p* < .001; [L E W U]: *F*(3, 177) = 4.73, *p* = .003; [U L E W]: *F*(3, 177) = 1.91, *p* = .003).
